# Internet-based cognitive behavior therapy for eating disorders – Development and feasibility evaluation

**DOI:** 10.1016/j.invent.2022.100570

**Published:** 2022-08-30

**Authors:** Anne-Charlotte Wiberg, Ata Ghaderi, Hanna Broberg Danielsson, Kousha Safarzadeh, Thomas Parling, Per Carlbring, Magdalena Jansson, Elisabeth Welch

**Affiliations:** a Centre for Psychiatry Research, Department of Clinical Neuroscience, Karolinska Institutet, Stockholm Health Care Services, Stockholm County Council, Stockholm, Sweden; b Department of Clinical Neuroscience, Karolinska Institutet, Nobels väg 9, 17177 Stockholm, Sweden; c eHealth Institute, Linnaeus University, 391 82 Kalmar, Sweden; d Student Health Center, Lund University, Sandgatan 3, 22350 Lund, Sweden; e Centre for Psychiatry Research, Department of Clinical Neuroscience, Karolinska Institutet, Stockholm Health Care Services, Stockholm County Council, Stockholm, Sweden; f Department of Psychology, Stockholm University, 106 91 Stockholm, Sweden; g Stockholm Center for Eating Disorders, Stockholm County Council, Wollmar Yxkullsgatan 27B, 118 50 Stockholm, Sweden

**Keywords:** Binge-eating, Enhanced cognitive behavioral therapy, Eating disorders, Feasibility, Internet-delivered therapy, User centered design

## Abstract

**Background:**

Eating disorders (ED) are severe psychiatric conditions, characterized by decreased quality of life and high mortality. However, only a minority of patients with ED seek care and very few receive treatment. Internet-delivered cognitive behavioral therapy (ICBT) has the potential to increase access to evidence-based treatments.

**Aims:**

The aims of the present study were to (1) develop and evaluate the usability of an Internet-delivered guided self-help treatment based on Enhanced Cognitive Behavioral Therapy (ICBT-E) for patients with full or subthreshold bulimia nervosa (BN) or binge eating disorder (BED) with a user centered design process, and (2) to evaluate its feasibility and preliminary outcome in a clinical environment.

**Method:**

The study was undertaken in two stages. In Stage I, a user-centered design approach was applied with iterative phases of prototype development and evaluation. Participants were eight clinicians and 30 individuals with current or previous history of ED. In Stage II, 41 patients with full or subthreshold BN or BED were recruited to a single-group open trial to evaluate the feasibility and preliminary outcome of ICBT-E. Primary outcome variables were diagnostic status and self-rated ED symptoms.

**Results:**

The user-centered design process was instrumental in the development of the ICBT-E, by contributing to improvements of the program and to the content being adapted to the needs and preferences of end-users. The overall usability of the program was found to be good. ICBT-E targets key maintaining factors in ED by introducing healthy eating patterns and addressing over-evaluation of weight and shape. The results indicate that ICBT-E, delivered in a clinical setting, is a feasible and promising treatment for full or subthreshold BN or BED, with a high level of acceptability observed and treatment completion of 73.2 %. Participation in ICBT-E was associated with significant symptom reductions in core ED symptomology, functional impairment as well as depressive symptoms, and the results were maintained at the 3-month follow-up.

**Conclusions:**

ICBT-E was developed with end-users' preferences in mind, in accordance with the identified recommendations, and the program was perceived as usable by end-users. The study demonstrated the potential of ICBT-E, which marks a step forward in the effort to make powerful, empirically supported psychological interventions targeting ED more widely available and accessible.

## Highlights

• Eating disorders are severe disorders but only a minority seek and receive care.
• Digital interventions have the potential to increase access to care.
• Internet-delivered cognitive behavioral therapy (ICBT-E) was developed.
• A user-centered design process was undertaken, contributing to improvements of ICBT-E.
• The feasibility and preliminary outcome of ICBT-E was evaluated with promising resuts.

## 1 Background

Eating disorders (ED) are among the most severe of all mental disorders as they are associated with significant psychiatric and medical comorbidity, increased mortality (van Hoeken and Hoek, 2020; Welch and Ghaderi, 2015) and impaired quality of life of both patients and their significant others (de la Rie et al., 2005; Jenkins et al., 2011). Moreover, EDs pose a substantial financial burden for afflicted individuals, their family and society as a whole (Agh et al., 2016; van Hoeken and Hoek, 2020). Epidemiological studies show that only a minority of patients with EDs seek professional treatment (Dobrescu et al., 2020; Hart et al., 2011), and when they do, few receive evidence-based treatment (Cooper and Bailey-Straebler, 2015). Consequently, there is an urgent need to improve access to evidence-based care.

Increased digitalization has had a transformative effect on delivery of mental healthcare. Digital innovations, such as internet-based cognitive behavioral therapy (ICBT), have generated new pathways to treatment with the potential to handle challenges related both to the patients not seeking treatment and to the health care system not being able to offer evidence-based treatment (Aardoom et al., 2016; Kazdin et al., 2017). It has been established that ICBT has similar effects as treatments performed face to face, both in the short term and long term (Andersson et al., 2018; Carlbring et al., 2018). A number of ICBT for EDs have been developed and, when evaluated, they have shown successful reduction of ED symptomatology (Carrard et al., 2011a; de Zwaan et al., 2017; Fitzsimmons-Craft et al., 2020; Ruwaard et al., 2013; Sanchez-Ortiz et al., 2011; Strandskov et al., 2017; ter Huurne et al., 2015; Wagner et al., 2016; Wagner et al., 2013; Zerwas et al., 2017) with small to large effect sizes (Schlegl et al., 2015). However, the full potential of digital interventions is hampered by poor user engagement and adherence to treatment, particularly evident in internet-based interventions (Linardon et al., 2018). Major barriers to the acceptance and use of new digital interventions are usability issues and that end-user needs have not been considered during the development (Borghouts et al., 2021; Torous et al., 2018). A well-established methodology that involves end-users in the development of products, such as digital interventions, is user-centered design (UCD; (Graham et al., 2019; Lyon and Koerner, 2016) where end-users are involved early and throughout the development process. Through iterative steps with prototypes and user evaluations, improved versions of the products are developed that are adapted to the needs and perceptions of the users, as well as to the contexts in which they will be used. UCD can contribute to the development of digital interventions that are functional, useable, acceptable and can promote the intended clinical outcome (Kushniruk and Nohr, 2016).

Digital interventions can also be designed in a way that can optimize clinical outcomes. According to a systematic review and meta-analysis about digital interventions for EDs, a varied range of multimedia formats, such as written text, pictures, graphics, animations, audio, and video (Barak et al., 2009) was associated with better treatment outcome (Barakat et al., 2019). Although it has been established that end-users prefer content delivery via video and graphics (Linardon et al., 2021), digital interventions are predominantly delivered via text-based content with few multimedia options, similar to written self-help manuals. Furthermore, the interactivity that the digital mode allows is often underutilized (Loucas et al., 2014).

Cognitive behavioral therapy (CBT) is recommended as the treatment of choice for bulimia nervosa (BN), binge eating disorder (BED) and related conditions (Hilbert et al., 2017; National Institute for Health and Care Excellence, 2017). The treatment has been refined and the latest version, Enhanced Cognitive Behavioral Therapy (CBT-E) involves more potent methods and strategies (Fairburn, 2008). By the end of treatment around 65 % of patients have been found to be in remission, but the number increases to about 70 % at follow-up after 60 weeks (Fairburn et al., 2015).

Thus, powerful, empirically supported psychological treatments that target ED exist, but they do not reach those affected (Cooper and Bailey-Straebler, 2015). ICBT might increase access to treatment for a larger proportion of those who suffer from ED. It is critical to facilitate the user engagement and adherence to new digital interventions by ensuring their usability and involving end-users' needs and perceptions in the development. Studies are needed that take these factors into account (NICE, 2021), including studies in the field of ED (Graham et al., 2019; Linardon et al., 2020a). In addition, most empirically evaluated internet-based treatments for ED are based on early cognitive behavioral therapy for BN (Fairburn, 1981) and the feasibility and effectiveness of internet-based CBT-E remains an empirical question in need of investigation (Loucas et al., 2014).

## 2 Aims

The aims of the present study were to (1) develop and evaluate the usability of a guided internet-based CBT-E (ICBT-E) for patients with full or subthreshold bulimia nervosa (BN) or binge eating disorder (BED) with a UCD process, and (2) to evaluate the feasibility and preliminary outcome of ICBT-E in a clinical environment.

The following research questions formed the basis for the design of the project:

What was the experience and perception of the end users of the usability of ICBT-E?
Is ICBT-E a feasible method for treating patients with full or subthreshold BN or BED in a clinical setting?
Does ICBT-E lead to reduction in core ED symptomology, functional impairment and depressive symptoms from pre- to post-intervention?
Will potential symptom reductions be maintained at a 3-month follow-up?

## 3 Methods

This study was undertaken in two stages. In the first, Stage I, ICBT-E was developed and evaluated with a user-centered design process. Subsequently, in Stage II the feasibility and preliminary outcome of ICBT-E were evaluated. The study was approved by the Swedish Ethical Review Authority (2018/1221-31/5).

### 3.1 Stage I: development of ICBT-E

The design and development processes were based on a multidisciplinary and user-centred design approach (Graham et al., 2019). The international standard for usability, ISO 9241-11 (2018) and the definition of usability by Nielsen (1993) was used in this study to provide guidance on usability. The ISO 9241-11 describes usability as “the extent to which a product can be used by specified users to achieve specified goals with effectiveness, efficiency, and satisfaction in a specified context of use” (ISO, 2018). According to Nielsen, usability includes the five quality components: learnability, efficiency, memorability, errors and satisfaction (Nielsen, 1993).

#### 3.1.1 Participants and setting

The project team for multidisciplinary collaboration consisted of researchers from three universities with different research backgrounds covering psychiatry, psychology, and health informatics and clinicians working with the treatment of EDs at the ED clinic Stockholm Center for Eating Disorders (SCED), Stockholm County council, in Sweden. Study participants were patients and individuals with current or previous history of ED (*n* = 30), who were recruited from the outpatient unit at SCED and from a patient organization. The inclusion criteria for participation were adults with current or lifetime diagnoses of EDs. In addition, clinicians working in the outpatient unit at SCED were recruited (*n* = 8). Informed consent was obtained from all participants before participation.

#### 3.1.2 Data collection

Focus groups were conducted with clinicians, patients and individuals from the patient organization. The interview guide that was used was semi-structured and contained questions about usability and how the content was presented in the digital format (see online supplement). In addition, Think Aloud observations were carried out to assess usability and to further understand the end-user needs. Think Aloud is a method in which the participants are observed while performing predetermined tasks and where they concurrently express their thoughts and reactions. The method is commonly used in usability studies. It provides extensive, detailed data and only a small sample of five to eight users is needed to identify 80–85 % of usability issues (Nielsen, 1994). Focus groups were audio recorded and Think Aloud observations were audio and video recorded using screen-capture software.

Usability was also evaluated through the System Usability Scale (SUS; (Brooke, 1996), which measures usability based on ISO 9241-11 (2018).

#### 3.1.3 Development process

The UCD process, was carried out in accordance with the recommendations of Graham et al. (2019) and consisted of the following eight phases 1) Development of a prototype, 2) Evaluation, 3) Refine and develop, 4) Evaluation, 5) Refine and develop, implementation, 6) Ongoing feedback collected from users, 7) Refine and develop, and finally 8) Validate. The clinical content was written and structured by researchers and clinicians. The written content was entered into the platform together with pictures and graphics. To optimize interactivity, access and efficiency, most of the content was delivered as films and infographics. In addition, exercises, self-assessment and self-monitoring were developed, based on the CBT-E treatment model. The final version of ICBT-E was produced after feedback from repeated focus group interviews (four in total) and 16 Think Aloud observations as well as a validation of the usability by 24 patients who had used the treatment for approximately 5 weeks.

#### 3.1.4 Data analysis

Screen- and audio recordings from focus groups and Think Aloud observations were transcribed by two transcribers. The goal of the analyses was to generate insights that would enable refinement of ICBT-E. Transcription was therefore not performed verbatim, which thereby allowed for a more rapid processing (Mann et al., 2018).

For the thematic analysis, transcripts were reviewed and transcribed verbatim, including participants' nonverbal behavior. The thematic analysis was conducted according to Braun and Clarke's model (Braun and Clarke, 2006), using both an inductive and deductive approach. Transcripts were read multiple times by two authors (AW and MJ) to familiarize themselves with and to gain an overview of the entire material. All transcripts were coded by AW and 60 % of the transcripts were independently double-coded by AW and MJ to ensure consistency. Coded data was sorted by AW and MJ into tentative themes, based on semantic level. The themes were reviewed, which generated modified and newfound themes, to enable a narrative for the findings that was representative of the data. The themes were given final names, consistent with their content. Regular meetings with the research team (EW, AG, HBD, AW, MJ) were held throughout the entire process, to discuss and refine the analyses. NVivo 12 software package, produced by QSR International, was used for coding and analysis.

### 3.2 Stage II: feasibility and preliminary outcome of ICBT-E

#### 3.2.1 Design

A single-group open trial was conducted to examine the feasibility and preliminary outcome of ICBT-E. Clinical outcome data was collected on three occasions; at pre- treatment, at post-treatment and at 3-month follow-up.

#### 3.2.2 Participants

All patients seeking treatment at the Stockholm Centre for eating disorders (SCED) meeting the criteria for full or subthreshold BN or BED according to DSM-5 (American Psychiatric Association, 2013) were offered participation in the study until a total of 50 patients were recruited. Patients were excluded due to pregnancy, concurrent alcohol or substance abuse, psychotic and/or suicidal symptoms in the absence of qualified care, severe problems with reading and writing and/or any other severe psychological and/or physical impairment that could hinder participation. Diagnosis were initially assessed by well-trained clinicians using the Mini International Neuropsychiatric Interview (MINI; (Sheehan et al., 1998) according to local practice at SCED, and then verified by the SCID-5 interview (First et al., 2016) conducted by a trained research nurse. Data collection was initiated on June 6th, 2019 and terminated on June 16th 2021. The characteristics of the patients are presented in Table 1.

**Table 1:** The characteristics of the participants.

	*M*(*SD*; range)
Age	34.5(10.1; 19–61)
Gender	*N*(%)
Female	39(95.1)
Male	2(4.9)
Education level	
Nine-year compulsory school	2(4.9)
High school	14(34.1)
University and above	25(61)
Occupation	
Student	10(24.4)
Employed	29(70.7)
Self-employed	1(2.4)
Long-term sick leave	1(2.4)
Diagnosis	
BN	23(56.1)
BED	12(29.3)
Subthreshold BN or BED	6(14.6)

Notes: Values presented outside of parenthesis = number of participants, Values presented within parenthesis = percentage of participants, BN = Bulimia Nervosa, BED = Binge Eating Disorder.

#### 3.2.3 Measures

##### 3.2.3.1 Feasibility of ICBT-E

The feasibility assessment included quantitative measures of rates of recruitment, treatment acceptability, treatment credibility and expectancy, treatment adherence and occurrence of negative effects due to treatment.

Feasibility of the study procedures was determined based on recruitment to the study, and retention to follow-up at the endpoint (3 months).

Acceptability of ICBT-E was assessed at post-treatment, with a visual analog scale (VAS) from 0 (representing minimal acceptability) to 10 (representing maximum acceptability).

An adapted version of the Credibility/Expectancy Questionnaire (CEQ) (Devilly and Borkovec, 2000) was used to evaluate credibility and expectancy. The CEQ has demonstrated adequate psychometric properties with regard to internal consistency, inter-item correlations and test-rest reliability (Devilly and Borkovec, 2000).

Adherence was calculated as completers, i.e., patients who completed 80 % or more of the chapters. The mean number of completed chapters was also coded.

Occurrence of negative effects due to treatment was assessed using The Negative Effects Questionnaire (NEQ) - 20-item version (Rozental et al., 2019b). The NEQ is a self-administered measure of negative effects and differentiates between negative effects that are attributed to treatment and those possibly caused by other circumstances. The items are distributed across five factors: symptoms, quality, dependency, stigma and hopelessness.

Participants also completed a socio-demographic questionnaire detailing age, computer literacy, education, employment status, income and residence area at baseline.

##### 3.2.3.2 Primary outcome measures

Primary outcome variables included the clinical presentation of full or subthreshold BN or BED according to DSM-5 assessed by The SCID-5 (First et al., 2016), and key behavioral features as well as core psychopathology of EDs measured by the Eating Disorders Examination Questionnaire (EDE-Q; (Fairburn and Beglin, 2008).

*The SCID-5* (First et al., 2016) is a semi-structured interview instrument for assessment of current and lifetime of psychopathology according to DSM-5. In this study, the Research Version of the SCID-5 (module H) was used in order to assess and establish ED diagnosis.

*The EDE-Q (6.0)* (Fairburn and Beglin, 2008) measures the core pathology of EDs, conceptualized as the excessive importance of weight and shape in determining self-worth, as well as the frequency of core ED behaviors, including binge eating and compensatory behaviors. It provides a global score as well as four subscales: Restraint, Eating Concern, Shape Concern and Weight Concern. Initial and later reports indicate satisfactory psychometric properties such as concurrent validity, internal consistency, temporal stability and test-retest reliability (Fairburn and Beglin, 1994; Welch et al., 2011).

##### 3.2.3.3 Secondary outcome measures

*The Clinical Impairment Assessment (CIA) (3.0)* (Bohn et al., 2008) is a self-report measure developed to assess the psychosocial impairment secondary to ED features. CIA has shown satisfactory psychometric properties in terms of internal consistency, test-rest reliability, construct and discriminant validity and sensitivity to change (Bohn et al., 2008; Welch et al., 2011).

*The Patient Health Questionnaire (PHQ-9)* (Kroenke et al., 2001) is a brief measure used to screen for depression. The internal reliability of the questionnaire as well as the test-retest reliability have been found to be satisfactory (Kroenke et al., 2001).

#### 3.2.4 Procedure

Patients who were recruited to internet-based treatment after a standardized basic assessment according to local practice at SCED were invited to participate in the study by clinicians. Participants received written and verbal information about the study with the opportunity to ask questions, and prior to inclusion they signed an informed consent. This was followed by the SCID diagnostic interview, conducted by a trained research nurse by telephone, in order to confirm the diagnosis, to ensure that participants met the inclusion criteria for participation and did not meet any exclusion criteria for participation. Subsequently, participants completed baseline self-report measures online. At the end of the treatment (after 15 weeks) and at 3 months' follow-up, the research nurse conducted a SCID diagnostic interview again with participants by telephone and self-report online measures were completed. The patients were consecutively recruited from the outpatient unit for adults at the SCED, from May 2019 until October 2020.

#### 3.2.5 Data analysis

All the statistical analyses were conducted in SPSS version 26. Data were initially screened by means of descriptive statistics. To test the normality of outcome variable, descriptive statistics and graphic presentations (e.g., histogram distribution and quantile-quantile (Q-Q) plots) were used. Treatment outcome was analyzed using generalized linear mixed modeling (GLMM), with a fixed effect of time and repeated observations nested within subjects, and random intercept, with the except of the EDE-Q weight concern, for which the random effect was excluded as the model did not converge with a random effect. Descriptive statistics and Q-Q plots showed reasonably normal distribution of most outcome variables and normal distribution and an identity link were thus specified in the analysis. For binge eating episodes, a Poisson distribution with a log link was used. The GLMM can effectively handle non-normal outcome distributions, and mixed models are inherently intention-to-treat. The proportions of participants who completed post- and follow-up-assessment in this study were 58–78 % and 70–74 % respectively. Within-group effect sizes were also calculated using Cohen's *d.*

Remission from ED at post-treatment and 3-month follow-up was established by the SCID-5 diagnostic interview. Full remission was defined as no symptoms or criteria being met for a sustained period of time. Partial remission was defined as only some remaining symptoms, compared to meeting the criteria for a full diagnosis of ED at baseline. Duration criterion for partial remission and full remission was three months. Additionally, to obtain full remission, EDE-Q global score had to be within one standard deviation (*SD*) from the general population mean (≤2.83) (Welch et al., 2011).

Furthermore, in order to assess whether the observed change in EDE-Q Global score among the participants were of clinical significance, the two-step criterion for clinically significant change, proposed by Jacobson and Truax (1991) was used. The Reliable Change Index (RCI) was first calculated to ensure that the observed change was not related to measurement errors. A reliable change had occurred if RCI was >1.96, which corresponds to a 95 % confidence interval. In the next step, a cut-off score was determined for the clinical group and the non-clinical group in accordance with the commonly used criterion for clinically significant change, EDE-Q global score within one *SD* from the general population mean (Fairburn et al., 2015). Participants whose mean EDE-Q global score after treatment had crossed the clinical cut-off score of ≤2.83 had a clinically significant change (Welch et al., 2011). Participants who passed the cut-off and reached a reliable change were considered to be recovered. Those who scored below the cut-off but did not reach reliable change were considered to be non-reliably recovered. Participants who reached reliable change but did not pass the cut-off were considered to be improved, while those who neither passed the cut-off nor achieved reliable change were considered to be unchanged. Participants who achieved reliable change in the opposite direction were considered to be deteriorated.

## 4 Results

### 4.1 Stage I: Development of ICBT-E

#### 4.1.1 Participant characteristics

In total, 14 individuals participated in focus groups and Think aloud observations. Six participants were individuals with the experience of an ED and 8 were clinicians at SCED. Participants age ranged from 30 to 62 with a mean age of 43.3 (*SD* = 10.1). There were 3 males and 11 females. One participant was a university student and 13 had completed university education. Usability evaluation through SUS was performed by 24 patients, ranging in age from 19 to 57 with a mean age of 35.1 (*SD* = 12.5). Two were males and 22 were females. One of these participants had completed nine-year compulsory school, 9 had completed high school and 14 had completed university education.

#### 4.1.2 Qualitative results

The guidance of ISO 9241-11 (2018) and Nielsen's definition of usability (Nielsen, 1993) during the UCD process contributed to identification and correction of usability issues, grammatical errors and technical bugs, and the final version of ICBT-E was found to be usable. The UCD approach also enabled the content to be adapted to the users' needs and preferences. The thematic analysis generated six overarching themes: *Accessibility, Navigation, Design, Transformation of Information, Patient-Facilitator Interaction and Elements of Interventions*.

The theme *Accessibility* reflected the fact that participants appreciated the opportunity to use the treatment after it had ended. They highlighted the need to be able to log in easily to the treatment, because registrations are carried out several times a day. Login with BankID, a national digital identification service, was experienced as inefficient by some participants and several expressed the idea that registration in an app would be preferable:

“I think that […] I probably should have used an app myself, that I download from the Internet, and where you can record food and then fill out later. Because it's pretty complicated to log on […] with BankID every time. Especially when you are going to do it several times a day”.

Regarding *Navigation*, the majority of participants found it easy to navigate through the program. Some had difficulty in understanding the structure initially, but learned it after a short time of use, during the Think Aloud observations:

“Now I'm beginning to learn this, how it works here. Meal plan” (finds right away). “Instruction, right.”

(Fills in)

However, a usability issue that was observed was that participants had difficulty navigating between Modules, Chapters and Forms, which was addressed with a clarified structure for headings.

Comments on the program's graphic *Design* were mostly positive. However, a usability problem in relation to interaction design of self-monitoring of meals emerged. The initial self-monitoring form gave a deficient overview of the eating and was redesigned in several rounds. The final version was designed with a timeline that allowed registration of all meals as they occurred during the day:

“So, when we do it on paper they fill it out, then it is possible to put it in like, in the right place in time or what to say. Here it's like something else, it will be something else. Like something outside.”

Within the theme *Transformation of Information*, it became clear that the majority of participants appreciated that the content was largely presented via film, although some stated that they assimilate information better via text. This resulted in each film being provided with a PDF file with a summary of the film's content in text:

“I thought it seemed maybe a little too long and too much information, and then I thought oh, such good information! I would like to have this in writing, I thought.”

The *Patient-Facilitator Interaction*, via email messages in the platform, was perceived as valuable, as was the facilitator's insight into the patient's work with the various exercises in the program. Comments on the communication between patient and facilitator concerned whether it should be related to the content of the treatment or to other areas of life. The majority of participants favored the communication being related to the content of the treatment:

“As a patient I also think it's good because this is what you need to focus on. And then I think that it is easier to float off somewhere if this is given, that is, if you are given the opportunity to do so. I think it's good that it's controlled, that this is what we do.”

Feedback within the theme *Elements of Interventions* included the opinion that the Problem-solving exercise could be difficult to understand, which resulted in a new film with a patient and therapist going through Problem-solving together. One intervention that several participants reported as difficult to do independently at home was weekly weighing, but the participants felt that the content of the film and text could support carrying out the exercise:

“Yes, but ’I haven't weighed myself in such a long time, and I go a little crazy when I weigh myself, I haven't managed to do this', a little elevated anxiety. But this is a very good film, that explains it and I guess that you also get some kind of graph that helps you see that a fluctuating weight is a stable weight.”

The most central feedback from users during Phase 6 (ongoing feedback collected from users), which was not included in the thematic analysis, concerned the self-monitoring form. Although the form was refined in several rounds, some users reported usability issues related to its presentation in mobile phones. These users perceived that the self-monitoring form required too much scrolling, the overview of meals was deficient and it was difficult to enter the information in the text fields. However, the functions included in the treatment software platform did not enable the design of a self-monitoring form with optimal usability. Therefore, the form was not redesigned in phase 7. Consequently, it was left open for patients to digitally self-monitor their meals or to use traditional pen-and-paper self-monitoring.

#### 4.1.3 System Usability Scale

A total of 24 patients responded to the System usability scale, with a mean score of 76.46 (*SD* 14.20; range 47.50–100.00). Thus, the usability of the program was within the acceptable range for better systems (Bangor et al., 2008).

#### 4.1.4 The internet-based treatment NÄRA

Table 2 presents the structure and content of the ICBT-E program (named NÄRA).

**Table 2:** Overview of the treatment*.*

Week	Module	Chapter	Content
1	1	1: Preparing for treatment	- Education about the treatment and EDs. - Interventions to help patients decide to change.
2	2	2: Starting well and achieving early change	- Education about ED maintenance. - Creating a personalised case formulation. - Establishing real-time self-monitoring of food intake. - Education about body weight, weight change and support to begin to perform weekly weighing at home.
3,4		3: Regular eating	- Education about regular eating. - Interventions to establishing a pattern of regular eating and meal plan.
5		4: Problem behaviors and alternative activities	- Interventions to identify problem behaviors. - Introducing alternative activities (i.e. helpful substitute behaviors in replacement for problem behaviors).
6		5: Problem solving	- Education about the proactive problem-solving technique. - Practice of proactive problem-solving skills.
7	3	6: Taking stock and planning for the rest of the treatment	- Review of progress and engagement within the treatment. - Identifying barriers to change - Review and revision of case formulation - Identifying problems to be addressed in module 4.
8	4	7: Self-evaluation	- Education about self-evaluation. - Creating the self-evaluation, represented by the pie chart. - Identifying new personal domains of self-evaluation. - Interventions to identify activities or areas of life that the patient wants to engage in.
9		8: Dietary restraint & rules, and control over eating	- Education about dietary restraint, dietary rules and control over eating. - Identifying and breaking remaining dietary rules. - Identifying and reintroducing avoided food. - Engaging in new activities or areas of life.
10,11		9: Problems with body image	- Education about over-evaluation of shape and weight and its consequences. - Monitoring body-checking behaviors, body avoidance and feeling fat. - Interventions to reduce body-checking behaviors, body avoidance and feeling fat. - Engaging in new activities or areas of life.
12	5	10. Ending well	- Education about realistic expectation about time after treatment, and prevention and handling relapse. - Review of progress - Review and revision of case formulation - Identify remaining ED features and plan for how these will be addressed - Formulation of a maintenance- and relapse prevention plan.

The duration of the treatment is 12–15 weeks and consists of five modules which by large correspond to the four stages in CBT-E. Patients are instructed to spend at least 30 min every day working with the treatment content. The facilitator logs on once a week to review the patient's exercises, and provides feedback, with the role of supporting and guiding the patient to proceed with the treatment.

The initial treatment interventions include introduction, psychoeducation, case-formulation, daily self-monitoring of food-intake, establishing eating plans and regular eating patterns, weekly weighing, alternative activities (i.e. meaningful behaviors that are incompatible with disordered eating behaviors), and problem solving. These interventions are followed up by an in depth evaluation of the patient's progress (i.e., the second phase of CBT-E). Issues considered together with the patient include the patients' engagement in ICBT-E, commitment to the treatment plan, external circumstances that may hinder or enhance progress and so forth. At this point, termination of ICBT-E, or transfer to other treatment options might be considered in light of inadequate progress. In the latter part of the ICBT-E, effort is devoted to the topics of self-esteem, addressing all forms of dieting, and shape and weight concerns. The patient is encouraged to engage in activities that are completely independent of food-intake, shape or weight, and incompatible with disordered eating behavior. Dietary rules and rigid eating habits are identified and revisited, and finally, issues concerning shape and weight are addressed by identifying and modifying unhelpful behaviors and thoughts. The treatment is terminated with the enactment of a maintenance plan detailing progress, maintenance of gains and future hurdles.

After completion of treatment, the patient has access to the treatment content for six months, without support or facilitation by a facilitator. Three months after the end of treatment, a follow-up session is conducted via video.

### 4.2 Stage II: Feasibility and preliminary outcome of ICBT-E

#### 4.2.1 Study flow and sample characteristics

**Fig. 1:**
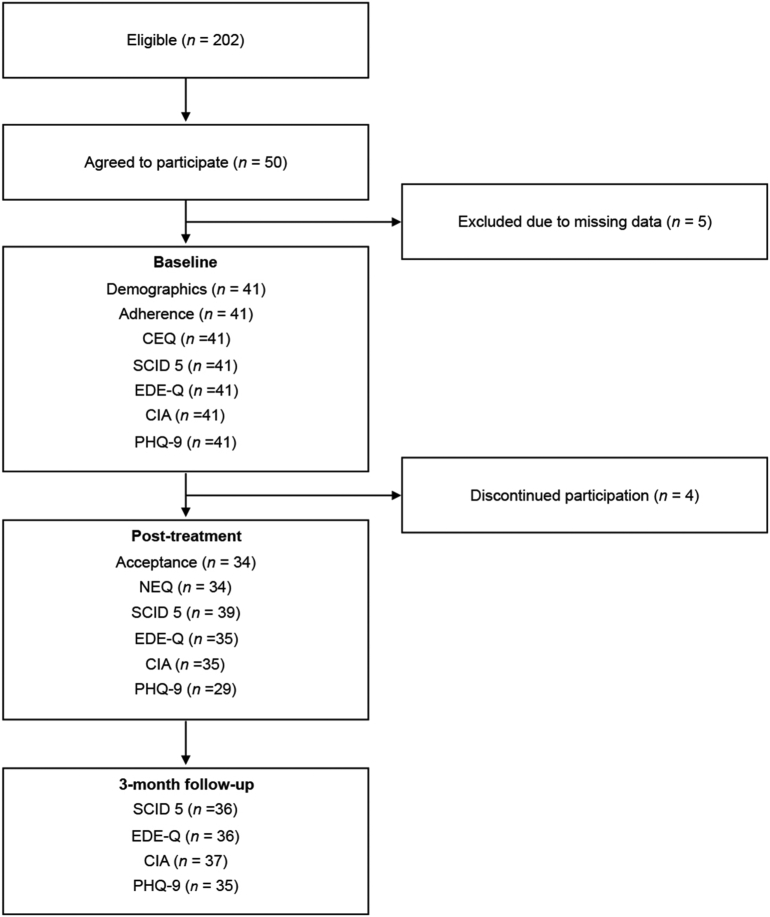
Study flow.

Fig. 1 shows the study flow. During this period, a total of 202 patients with full or subthreshold BN or BED according to the DSM-5 started the ICBT-E. Of those 202 patients, 50 (25 %) agreed to participate in the study, 5 (10 %) were excluded due to missing data at baseline, 4 (8 %) withdrew from the study and 35–37 (70–74 %) completed all or portions of the follow-up assessments at the endpoint. The patients were recruited from 05/2019 until 10/2020. The target sample size (*n* = 50) was recruited with a mean rate of 3.1 participants per month for the 16-month recruitment period. The characteristics of the patients are presented in

Table 1.

#### 4.2.2 Feasibility

The mean score based on the visual analog scale (0−10) for the acceptability of ICBT-E was 7.76 (*SD* = 1.86). Credibility and expectancy ratings on the two rating scales, ranging from 1 to 9 and from 0 to 100 %, showed moderate to high overall rating. In terms of how credible the treatment was perceived, the mean ratings were 7.02 (*SD* = 1.77) (how logical does the intervention offered to you seem?), 6.27 (*SD* = 1.60) (how successfully do you think this intervention will be in reducing your eating disorder symptoms?) and 6.41 (*SD* = 1.86) (how confident would you be in recommending this intervention to a friend who experiences similar problems?). The mean ratings of expectancy were 66.34 (*SD* = 20.71) (by the end of the treatment, how much improvement in your symptoms do you think will occur?), 6.07 (*SD* = 1.89) (how much do you really feel that the intervention will help you reduce your eating disorder symptoms?) and 62.44 (*SD* = 22.89) (by the end of the treatment, how much improvement in your symptoms do you really feel will occur?).

Of the 41 participants, 30 (73.2 %) completed the treatment (defined as completion of at least 80 % of the chapters). The mean number of completed chapters was 8.68 (*SD* = 2.06) out of 10 chapters. The reasons for dropping out among the 11 participants who did not complete the treatment were the need for higher level of care (*n* = 5), personal or familial circumstances (*n* = 4), low motivation to change (*n* = 1), or unknown reason (*n* = 1).

Negative effects due to treatment were noted as measured by the NEQ, with a frequency of negative effects from treatment ranging from 0 to 13, with a mean of 3.62 (*SD* = 2.98) (score range: 0–20). Most adverse effects included an increase in symptoms due to treatment, which 24 (70.6 %) participants experienced. The most frequently reported question was “I experienced more anxiety”, reported by 15 (44.1 %) participants. The total negative impact of treatment (i.e., how impactful each patient viewed any experienced negative effects) varied greatly, ranging from 0 to 33 with a mean of 7.12 (*SD* = 7.41) (score range: 0–80).

#### 4.2.3 Treatment outcome

**Fig. 2:**
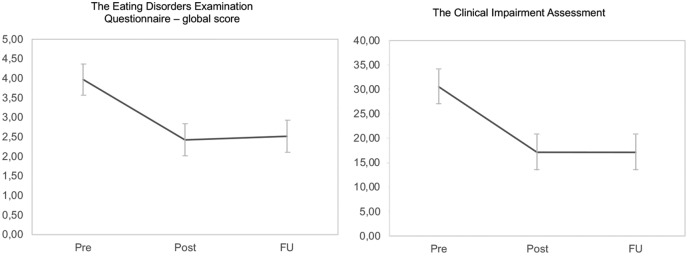
Outcome on the EDE-Q global score and the CIA at pre- post-, and at three months follow up.

Significant reductions in core ED symptomology, functional impairment as well as depressive symptoms were noted from baseline to end of the treatment. The results were maintained at follow-up. Fig. 2 depict overviews of the clinical outcome data. GLMM-estimated means and standard errors of the outcome variables and time points are presented in Table 3.

**Table 3:** Estimated means (standard errors) and Cohen's d of the outcome variables (negative effect sizes indicate deterioration).

Measure	Estimated means (standard errors)			Effect size (Cohen's d)		
	Pretest *M(SE)*	Posttest *M(SE)*	3-month follow up *M(SE)*	Pre to post	Post to 3-month follow up	Pre to 3-month follow up
EDE-Q						
Global score	3.97(0.20)	2.42(0.21)	2.52(0.21)	1.25	−0.08	1.17
Restraint	3.39(0.25)	1.55(0.26)	1.73(0.26)	1.20	- 0.12	1.08
Eating concern	3.41(0.23)	1.76(0.24)	1.68(0.24)	1.16	0.06	1.21
Shape concern	4.91(0.23)	3.42(0.24)	3.55(0.24)	0.48	−0.10	0.39
Weight concern	4.22(0.21)	2.95(0.21)	3.15(0.22)	0.99	−0.16	0.83
OBE	11.26(1.38)	3.57(0.68)	3.27(0.63)	1.11	0.08	1.17
CIA	30.54(1.78)	17.14(1.87)	17.18(1.84)	1.21	0.00	1.20
PHQ-9	12.51(1.00)	8.95(1.11)	9.40(1.05)	0.58	−0.07	0.50

*Note:* EDE-Q global score = global score for The Eating Disorders Examination Questionnaire, EDE-Q restraint = restraint subscale, EDE-Q eating concern = eating concern subscale, EDE-Q shape concern = shape concern subscale, EDE-Q weight concern = weight concern subscale, EDE-Q OBE = frequency of objective binge eating episodes, CIA = Clinical Impairment Assessment, PHQ-9 = Patient Health Questionnaire.

Results from the GLMM-analysis showed significant time effects for EDE-Q global score and its subscales: EDE-Q global score (*F*(2, 109) = 56.23, *p* < .001), Restraint (*F*(2, 109) = 39.08, *p* < .001), Eating concern (*F*(2, 109) = 34.91, *p* < .001), Shape concern (*F*(2, 109) = 32.34, *p* < .001) and Weight concern (*F*(2, 109) = 28.99, *p* < .001). Significant time effects for objective binge eating episodes reported in EDE-Q was also found (*F*(2, 109) = 34.16, *p* < .001). The main effect of time was accounted by changes from pre- to post-treatment, reflected in non-overlap of the 95 % confidence interval of the mean for pre- versus post-treatment, while the 95 % confidence interval for the treatment assessment at post-treatment and 3-month follow up were basically identical. At post-treatment, abstinence rates from binge eating episodes assessed by EDE-Q for the last 28 days, was 28.6 % (*n* = 10). Among the participants, 46.3 % (*n* = 19) reported self-induced vomiting at baseline and abstinence rate post-treatment was 74.3 % (*n* = 26). At the 3-month follow-up, the abstinence rates from binge eating increased to 44.4 % (*n* = 16). Abstinence rates from self-induced vomiting increased to 80.6 % (*n* = 29).

In terms of remission from ED diagnosis, 48.7 % (*n* = 19) of the participants reached partial remission at post treatment and 7.7 % (*n* = 3) reached full remission. At 3-month follow up, 38.9 % (*n* = 14) were in partial remission whereas 19.4 % (*n* = 7) were in full remission.

**Fig. 3:**
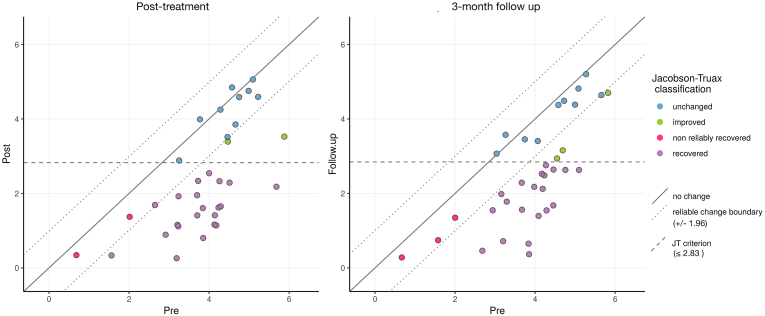
Jacobson-Truax plot of clinically significant change at post-treatment and at 3-month follow-up. *Note.* Post-treatment: *n* = 35, 3-month follow up: *n* = 36.

Fig. 3 presents the proportion of participants who were recovered, non-reliably recovered, improved or unchanged at post-treatment and at 3-month follow-up. At post-treatment, 60 % (*n* = 21) of participants were recovered, 5.7 % (*n* = 2) were non-reliably recovered and 5.7 % (*n* = 2) were improved. The proportion of participants who were unchanged was 28.6 % (*n* = 10). At 3-month follow up, 55.6 % (*n* = 20) were recovered, 8.3 % (*n* = 3) were non-reliably recovered, 8.3 % (*n* = 3) were improved and 27.8 % (*n* = 10) were unchanged. No participant deteriorated between baseline and follow-up.

The GLMM-analyses also showed a significant decrease in psychosocial impairment due to ED across time, measured by CIA: (*F*(2,110) = 38.21, *p* < .001) and a significant decrease in depression measured with the PHQ-9: (*F*(2,102) = 7.85, *p* = .001), with the main effect of time observed from pre- to post treatment.

## 5 Discussion

This study describes the multidisciplinary, user-centered design process of guided ICBT-E for patients with full or subthreshold BN or BED. The study also presents findings from the evaluation of the feasibility and preliminary outcome of the treatment in a clinical setting.

### 5.1 Development of ICBT-E

The UCD process of ICBT-E included a total of eight phases with development, evaluation, ongoing feedback after implementation, refinement and validation (Graham et al., 2019), and contributed to improvements and adaptions of the program, according to the needs and perceptions of end-users. User feedback and improvements were described within the six overarching theme, Accessibility, Navigation, Design, Transformation of information, Patient-Facilitator Interaction and Elements of interventions.

Based on Nielsens five quality components, learnability, efficiency, memorability, errors and satisfaction (Nielsen, 1993) the final version of ICBT-E was considered to be usable. The users could easily familiarize themselves with the program during the Think aloud observations. They could also easily navigate to and accomplish the planned tasks. No severe errors were detected and evaluation by focus groups and Think aloud showed that the participants were satisfied using the program. When the usability of the program was validated through the System Usability Scale (SUS), the usability was found to be within the acceptable range for good systems (Bangor et al., 2008). The multidisciplinary, user-centered design process resulted in a 12–15 week guided internet-based treatment, based on evidence-based treatment for EDs, CBT-E.

Contrary to many other ICBT for ED, the ICBT-E content in this study was delivered through various multimedia channels, with the majority of the content delivered via video and infographics, according to what individuals with EDs prefer (Linardon et al., 2021). Underutilization of interactive features in digital interventions and reliance on programs that are largely based on text, has in previous studies been suggested to affect the outcome of treatment (Nordgreen et al., 2019). Patients have also expressed that they dislike reading long texts in digital interventions and often skip over the content. (Nitsch et al., 2019). In addition, internet-delivered treatments are often developed and disseminated without evaluating usability, and it is currently recommended that aspects of usability are taken into account early on in the development of these treatments (Yogarajah et al., 2020). The present findings suggest that the multidisciplinary, user-centered design approach is a useful method for the development of digital interventions. Consequently, the prerequisites for use, engagement and clinical outcome are optimized so that the potential offered by digital interventions can be fully utilized (Barakat et al., 2019; Kushniruk and Nohr, 2016).

After implementation in the clinical setting, most feedback from end-users concerned the self-monitoring form of meals, which some patients chose not to use due to usability issues. This highlights the need for usability and user-centered design principles to be incorporated into the development of digital interventions intended for use in healthcare. To date, the technical functionality of the platform for ICBT-E does not allow the development of a smartphone application as an alternative to the self-monitoring of meals, as some participants requested, but various alternatives for improving the self-monitoring are being explored. However, since usability evaluated by SUS showed that the overall usability of ICBT-E was perceived to be good, and since the program has otherwise been adapted to the needs and preferences of end-users, the fact that some users perceived a lack of usability in the self-monitoring form of meals is not considered to be a barrier to the user engagement and adherence to ICBT-E.

### 5.2 Feasibility and preliminary outcome of ICBT-E

In summary, the results of this study indicate that ICBT-E is a feasible treatment of BN and BED as specified above. Participation in ICBT-E was associated with significant symptom reductions in core ED symptomology, functional impairment as well as depressive symptoms and the results were maintained at the 3-month follow-up.

The study mean recruitment rate of 3.1 participants per month was lower than anticipated. The clinic had long waiting times for treatment during the study period and the clinicians were therefore under pressure, which may have hampered recruitment of available patients. In addition, there was limited availability of facilitators and ICBT-E was a recently implemented method that facilitators were unfamiliar to use, which may also have contributed to a lower recruitment rate. With better conditions in terms of access to treatment for patients, access to facilitators and familiarity with the method, a future large-scale effectiveness trial of ICBT-E may be likely feasible.

ICBT-E was considered highly acceptable, with the mean score for acceptability on the VAS of 77.6 %. The treatment rational was rated as credible by the majority of the patients and they also expected the treatment to yield reasonable to substantial symptom reductions. These findings are encouraging, not only because it is adding to the overall feasibility of ICBT-E, but also because previous research identified low credibility and expectancy as predictors of failure to engage in ICBT for BN (Watson et al., 2017). In addition, perceiving ICBT as credible and expecting it to be effective has been associated with better adherence, treatment response and outcome (El Alaoui et al., 2015; El Alaoui et al., 2016). Treatment adherence was also satisfactory (73.2 %) compared to other internet-based interventions targeting EDs (57.6 %) (Schlegl et al., 2015), which is especially encouraging since treatment completion is lower in “real-world” trials (Byrne et al., 2016).

Some negative effects of treatment could be noted as measured by NEQ, with most negative effects reported in the domain of symptoms. Increased stress and anxiety in ICBT-E is expected as exposure to feared foods and body image exposure, is included in the treatment. Such increased stress and anxiety could be indicative of engagement in treatment, and can contribute to greater symptom reductions in the long run. In addition, reported frequency of negative effects, such as items that investigate increased symptoms due to the intervention, had in general none to negligible impact as rated by the patients themselves.

Most importantly, participation in ICBT-E was associated with significant and substantial reductions in core ED symptomology as measured by the EDE-Q with significant difference in means scores from pre-treatment to 3-months follow up. These findings were mirrored by the shift in representation of clinical diagnoses with notable decreases in representation of ED diagnosis and increases in remission and recovery from baseline to follow up. Of the participants, 58.3 % were in partial or full remission according to the SCID-diagnostic interview at 3-month follow-up. Based on the criterion by Jacobson and Truax (1991), 55.6 % of the participants, were recovered and additionally 8.3 % benefited from the treatment in terms of improvements. The results are encouraging and favorable compared to previous findings of internet-based intervention for ED showing clinically significant improvement related to EDE-Q of 39 % (Ruwaard et al., 2013) and of 45.7 % in BN symptoms (Carrard et al., 2011b). Direct comparisons are however complicated due to differences regarding study characteristics. At 3-month follow-up abstinence rates from binge eating and self-induced vomiting was 44.4 % and 80.6 % respectively. This is in line with other guided self-help studies for BED showing abstinence rates from binge eating of 40.4 % (Linardon, 2018), and favorable compared to guided self-help studies for BN showing abstinence rates from binge eating and purging of 31.6 % for treatment completers (Linardon and Wade, 2018). In a recent review by Pittock et al. (2018), abstinence rates from binge eating ± other behaviors were found to be 22.1 to 46.5 %.

Results were also significant with regard to the other outcome variables indicating sustainable reduction in functional impairment due to ED (CIA) and a decrease in depressive symptoms (PHQ-9), similar to other studies of internet-based interventions where impairment and depression decreased after ED-specific treatment (Dölemeyer et al., 2013; Wagner et al., 2016).

This study showcased the promising potential of ICBT-E, which marks a step forward in the endeavor to make evidence-based interventions for ED more widely available and accessible. Previous research has shown that a significant proportion of patients with EDs intend to use digital interventions for their ED symptoms, especially those with BN, and that a significant proportion even prefer digital interventions over face-to-face interventions (Linardon et al., 2020b). ICBT-E can bridge reported barriers to treatment such as experiences of shame and fear of stigma, geographic and financial barriers, limited access to specialized care and long waiting times for treatment (Ali et al., 2017; Kazdin et al., 2017), and provide treatment at an early stage. The benefits of early treatment are substantial for afflicted individuals, because early detection and treatment are among the most important factors for a good prognosis (Treasure et al., 2015). ICBT-E can also reduce costs for mental healthcare, as the digital format requires less resources, which enables the allocation of more resources to address complex cases.

### 5.3 Limitations and future studies

There are several limitations and areas that should be addressed in future research. One limitation of the study in Stage I was the homogeneity of the sample considering gender and education level. The majority of the participants had a high level of education and few men participated. Previous studies suggest that men benefit less from ICTB (Rozental et al., 2019a) and that less education predicts dropout from treatment (Watson et al., 2017). Inclusion of participants with lower education level and more men in the study could have shed light towards possible strategies that may have enhanced their adherence, engagement and outcome in treatment.

Furthermore, conclusions regarding the effects of ICBT-E warrants careful interpretation of the results, given the study design without control condition. Future randomized control trials (RCT) are needed in the effort to establish ICBT-E as an effective treatment that enjoys equivalent levels of evidence and legitimacy of its face-to-face counterpart CBT-E. Moreover, the vast majority of the sample consisted of well-educated females recruited from vicinity of Stockholm. This is not representative of the Swedish population that suffer from ED, let alone the wider global population of patients with ED, which most probably differ from this sample with regard to literacy, ethnicity, gender, culture, computer and internet proficiency. Although this is an initial feasibility study with no claims in generalizability, future research and replication studies are warranted in the pursuit to make ICBT-E more accessible to a wider audience.

Moreover, even though the majority of the participants benefited from ICBT-E, some of them remained unimproved during the treatment and follow-up. Given this, it is critical to inform healthcare providers about which patients that should be offered ICBT-E and which should be offered other treatment, by examining predictors and moderators of outcomes. In addition, testing a stepped care approach would be valuable since that could be a useful and cost-effective model for increasing patients' access to evidence-based psychological treatments (Nordgreen et al., 2016).

Patients with the diagnosis of AN were excluded which casts shade on a treatment that theoretically and at heart is a transdiagnostic approach to the treatment of ED. Future investigations into the effectiveness of ICBT-E in treating AN would be an appreciated contribution, since AN among adults admittedly is a condition that is particularly difficult to treat with an approximate recovery rate of only 25 % in clinical outcome studies (Treasure et al., 2020) and high attrition rates (Frostad et al., 2018).

Lastly, some limitations are related to conducting the study in a real-world clinical setting and its prevailing conditions, such as high volume of referrals to the clinic, long waiting times for assessment and delayed treatment which made randomization of patients infeasible. Nevertheless, conducting the study in a real-world clinical setting brought about the benefits that such entails, such as varying degrees of comorbidity and severity of EDs due to the study's few exclusion criteria, and different degrees of education and level of clinical experience of the therapists, which makes the results of the study representative of an unselected real-world population.

In summary, ICBT-E was developed with end-users' preferences in mind, in accordance with the identified recommendations, and the program is perceived as usable by end-users. The study provides preliminary evidence that ICBT-E, delivered in a clinical setting, is a feasible treatment with potential for promising outcomes for patients with BN, BED and subthreshold BN and BED.

## Funding

This work was supported by Vinnova. Vinnova had no role in study design, data collection, data analysis, interpretation of the data, in the writing of the report or in the decision to submit the paper for publication.

## Declaration of competing interest

The authors declare that they have no known competing financial interests or personal relationships that could have appeared to influence the work reported in this paper.

## Appendix A Supplementary data

Supplementary material Image 1
