# Supplementary material for: Internet-based cognitive behavior therapy for eating disorders – Development and feasibility evaluation
Source: Internet Interv. 2022 Aug 30;30:100570. doi: 10.1016/j.invent.2022.100570 (PMC9468502; doi:10.1016/j.invent.2022.100570)
Supplement: Supplementary file 1 — Supplementary material [file mmc1.docx]

**INTERVIEW GUIDE - FOCUS GROUPS**

**PRIOR TO FOCUS GROUP**

- Written informed consent from participants.
- Participants are assigned a login in for the program called NÄRA, two weeks before the focus group is conducted. They are encouraged to test the program via computer, smartphone and tablet.
- Collection of demographic questions.

**FOCUS GROUP SESSION**

Focus group process overview by moderator:

- The time set aside for the focus group interview.
- Review of purpose for the focus group and plans for using the results.
- Semi-structured interview guide: Some selected issues will be addressed, but issues that arise during the interview will also be discussed, guided by the moderator.
- Opportunity to sketch ideas and suggestions.
- The program will be open via screen for the opportunity to show and discuss the content during the interview.
- Ground rules and group norms for the focus group.

**EVALUATION PHASE 2**

**Interview guide**

1. Briefly introduction of participants.
2. You have had the opportunity to log in to the treatment program, how has it worked?
3. Have you experienced any technical difficulties with log in or using the treatment program?
4. How much of the program have you used?

*Moderator gives a brief overview of NÄRA.*

1. What is your overall impression of NÄRA?
2. How is the user introduced into the program? (missing/insufficient, sufficient, excellent)
3. How have you been able to orient yourselves within the program? (difficult to navigate/easy to navigate)
4. How do you perceive the program's design regarding, layout, color, headings etc?
5. Text (scarce, appropriate, voluminous)
6. Image, audio and video are missing (available on a small scale, available on a large scale)
7. Balance text and media (less good/missing, good, excellent)
8. How do you perceive the interactivity? (the interaction between the user and the program: input and feedback, forms, etc)

a) Forms (missing, available on a small scale, available on a large scale)

b) Tasks/worksheets (missing, available to a small extent, available to a large extent)

c) Feedback (unclear/missing, clear/sufficient, excellent)

1. Is any function difficult or cumbersome to use?
2. Is there any function/information/intervention that is missing?
3. Is there any function/information/intervention that is unnecessary?
4. Would you have developed any function/information/intervention in any other way?

*Assistant moderator summarizes information that has emerged.*

1. Do you recognize yourself in the summary? Need something emphasized or toned down?
2. Is there anything you want to add?

**EVALUATION PHASE 4**

**Interview guide**

*Moderator gives a brief overview of NÄRA.*

1. What is your overall impression of NÄRA from Chapter 6, “Taking stock and planning for the rest of the treatment”?

*Moderator goes through all the chapters*

1. Is any function difficult or cumbersome to use?
2. Would you have developed any function/information/intervention in any other way?
3. How is the user introduced into the program? (missing/insufficient, sufficient, excellent)
4. How have you been able to orient yourselves within the program? (difficult to navigate/easy to navigate)
5. How do you perceive the program's design regarding, layout, color and headings etc?
6. Text (scarce, appropriate, voluminous)
7. Image, audio and video are missing (available on a small scale, available on a large scale)
8. Balance text and media (less good/missing, good, excellent)
9. How do you perceive the interactivity? (the interaction between the user and the program: input and feedback, forms, etc)

a) Forms (missing, available on a small scale, available on a large scale)

b) Tasks/worksheets (missing, available to a small extent, available to a large extent)

c) Feedback (unclear/missing, clear/sufficient, excellent)

1. How do you perceive the communication in NÄRA? (possibility for the user to see the sender of information, contact healthcare provider/facilitator and clarity when information goes to another recipient - insufficient/sufficient/excellent)
2. Do patients get enough support in the program to proceed with the treatment? (contents in the chapters, interaction with the facilitator/interaction with the treatment program, notices of activities/ inactivity, obligatory exercises/forms, open and work sequentially with one module at a time)
3. How can facilitators further support the patient to proceed with the treatment?
4. How can facilitators support patients to address what is related to the content of NÄRA?

*Assistant moderator summarizes information that has emerged.*

1. Do you recognize yourself in the summary? Need something emphasized or toned down?
2. Is there anything you want to add?
